# Serological chemiluminescence immunoassay for the diagnosis of SARS‐CoV‐2 infection

**DOI:** 10.1002/jcla.23466

**Published:** 2020-07-16

**Authors:** Shao Lijia, Shen Lihong, Wang Huabin, Xu Xiaoping, Lu Xiaodong, Zhu Yixuan, He Pin, Xu Yina, Shan Xiaoyun, Wu Junqi

**Affiliations:** ^1^ Department of Clinical Laboratory Jinhua Municipal Central Hospital Jinhua China; ^2^ Department of Central Laboratory Jinhua Municipal Central Hospital Jinhua China

**Keywords:** antibody, COVID‐19, diagnostic value, SARS‐CoV‐2

## Abstract

**Objective:**

Dynamic monitoring of the concentration variation of IgM and IgG in patients with SARS‐CoV‐2 infections and exploring their diagnostic value for coronavirus disease‐19 (COVID‐19).

**Methods:**

A total of 15 patients with SARS‐CoV‐2 infection were enrolled as the COVID‐19 group, and 50 patients were enrolled as the control group. The concentrations of SARS‐CoV‐2‐specific antibodies (IgM and IgG) were detected by a chemiluminescence immunoassay (CLIA).

**Results:**

According to the cutoff value recommended by the manufacturer (cutoff = 10 AU/mL), the sensitivity, specificity, Youden index (YI), positive predictive value (PPV), and negative predictive value (NPV) of IgM were 60%, 100%, 60%, 100%, and 89.29%, respectively; and 86.67%, 100%, 86.67%, 100%, and 96.15%, respectively, for IgG. We reassessed the cutoff value of IgM. When the cutoff value for SARS‐CoV‐2 IgM was 1.83 AU/mL, the sensitivity, specificity, YI, PPV, and NPV were 93.33%, 98%, 91.33%, 93.33%, and 98%, respectively. During dynamic monitoring of the concentrations of IgM and IgG in COVID‐19 patients, we found the shortest times before a patient became IgM and IgG seropositive after symptom onset were 1.5 and 2 days, respectively. The longest times were 7 and 8 days, respectively. The positive rates of SARS‐CoV‐2 IgM and IgG both reached 100% in 8‐14 days after symptom onset.

**Conclusion:**

The IgM cutoff value of 1.83 AU/mL for the diagnosis of COVID‐19 was much better than the cutoff suggested by the manufacturer. SARS‐CoV‐2 infection can be ruled out if antibodies against SARS‐CoV‐2 are still undetectable 14 days after symptom onset.

## Graphical abstract

The new cutoff of IgM improve the diagnostic value. Considering the high diagnostic efficiency of IgG, we chose the day on which the SARS‐CoV‐2 IgG was close to 10.00 AU/mL. Then, we collected IgM data on the same day to select new cutoff values. When the cutoff value of IgM was 1.83 AU/mL, we obtained the maximum YI.

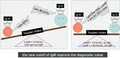

## 1 INTRODUCTION

The outbreak of the novel coronavirus disease (COVID‐19) quickly spread all over the world. As of March 31, 2020, the COVID‐19 disease has plagued over 190 countries, and over 700 000 people have been infected by SARS‐CoV‐2, which is currently spreading at alarming rates in Europe and the United States.^1^

SARS‐CoV‐2 is a new coronavirus belonging to the beta coronaviruses, with a single genus and a positive strand RNA.^2^, ^3^ In the past, six coronavirus species have been known to cause human diseases. HCoV‐229E, HCoV‐OC43, HCoV‐NL63, and HCoV‐HKU1 are only transmitted among human beings, and they cause relatively mild symptoms. However, the outbreak of severe acute respiratory syndrome coronavirus (SARS‐CoV) in 2002‐2003 and Middle East respiratory syndrome coronavirus (MERS‐CoV) in 2012 is zoonotic viruses that have caused pandemics of respiratory infections with high mortality.

SARS‐CoV‐2 is the seventh member of the newly discovered coronavirus species. It was reported that 81% of people with COVID‐19 have mild disease and never require hospitalization.^4^ The mortality of COVID‐19 in China was approximately 4.0%.^1^ However, the mortality of COVID‐19 among critically ill patients and those requiring mechanical ventilation was very high.^5^ Older age and a higher Sequential Organ Failure Assessment score on admission have been reported to be associated with high mortality.^6^ The virus is also reported to spread during asymptomatic phase, which greatly increases the difficulty of COVID‐19 disease prevention, diagnosis, and control of its spread. Therefore, many scientists and biological companies are committed to research and develop an accurate and rapid diagnostic test method to quickly identify a large number of symptomatic and asymptomatic in order to prevent and control virus transmission.^7^, ^8^

Real‐time reverse transcription PCR (RT‐PCR)‐based viral RNA detection is the gold standard for the diagnosis of COVID‐19.^9^, ^10^, ^11^, ^12^ The accuracy of RT‐PCR is heavily reliant on the sampling period and location. Missing the window period of viral replication can provide false‐negative results.^13^ It is highlighted that there is an urgent need to develop effective tools to recognize SARS‐CoV‐2‐infected patients.

It is widely accepted that IgM provides the first line of defense during viral infections, prior to the generation of adaptive, high‐affinity IgG secondary response that are important for long‐term immunity and immunological memory.^14^ Studies on SARS‐CoV and MERS‐CoV showed that antibodies were detectable in 80%‐100% of patients at 2 weeks after illness onset, and the antibodies can persist for at least 12 years.^15^, ^16^ Considering SARS‐CoV‐2 is the seventh member of the coronavirus species, it has 79.5% homology with SARS‐CoV. Serological detection of antibodies against SARS‐CoV‐2 provides another possibility for the early diagnosis of COVID‐19.

Chemiluminescence immunoassays (CLIA) are quantitative serological antibody detection assays, which have high sensitivity and specificity. The continuous detection of antibody concentrations could be used to assess the progression of COVID‐19 cases. Therefore, our research group is devoted to doing research on serological antibodies in SARS‐CoV‐2‐infected patients.

## 2 METHODS

### 2.1 Patients and samples

We enrolled 65 patients from Jinhua Municipal Central Hospital (from January 21, 2020, to March 5, 2020) because of their epidemiological history, signs, symptoms, and chest CT evidence, according to the National Health Commission of the People's Republic of China guidance (trial Sixth edition). Each subject consented for the SARS‐CoV‐2 nucleic acid detection. The 15 patients who tested positive for COVID‐19 were enrolled as the disease group. Meanwhile, the 50 patients who were excluded of having SARS‐CoV‐2 infection were enrolled as the control group. In order to analyze the diagnostic value of SARS‐CoV‐2 IgM and IgG for COVID‐19, 105 serum samples in total were collected from the two cohorts above. The study was approved by the Ethics Commission of Jinhua Municipal Central Hospital. Written informed consent was waived by the Ethics Commission of the designated hospital for emerging infectious diseases. The operations involved were carried out under strict biosafety conditions.

### 2.2 Main reagents and equipment

RT‐PCR for SARS‐CoV‐2 nucleic acid diagnostic reagents (Zhijiang, China); chemiluminescence test kit for SARS‐CoV‐2 (Yhlo, China); EX3600 nucleic acid automatic extraction equipment (Zhijiang, China); ABI 7500 real‐time fluorescent quantitative PCR equipment (Abi, USA); iFlash3000 automatic CLIA analyzer (Yhlo, China).

### 2.3 SARS‐CoV‐2 nucleic acid detection

RNA was obtained by fully automatic nucleic acid extraction equipment. The RT‐PCR system contains 5 μL RNA template + 19 μL SARS‐CoV‐2 nucleic acid detection mixture + 1 μL RT‐PCR enzyme; then, we used ABI 7500 real‐time fluorescence quantitative PCR equipment for amplification. The amplification conditions were as follows: 45°C 10 minutes; 95°C 3 minutes; 95°C 15 seconds; and 58°C 30 seconds, 45 cycles. Results: a. Positive: RdRP gene, N gene, E gene are all (+); RdRP gene (+) and N gene (+); RdRP gene (+) and E gene (+); If only RdRP gene (+), retested, and it is still only RdRP gene (+). b. Negative: RdRP gene, N gene, and E gene are all (−). c. Other near source coronavirus infection: N gene (+) or E gene (+).

### 2.4 Testing the concentration of SARS‐CoV‐2 IgM and IgG using the CLIA

Using an indirect two‐step immunoassay, the tests were conducted according to the procedures recommended by the manufacturer (Shenzhen Yhlo Biotech Co., Ltd). The resulting chemiluminescent reaction is measured as relative light units (RLUs). A direct relationship exists between the amount of anti‐SARS‐CoV‐2 IgM/G in the sample and the RLUs detected by the iFlash optical system. The results are determined via a calibration curve, which is an instrument‐specifically generated by 2‐point calibration and a master curve provided via the reagent QR code. The cutoff value of SARS‐CoV‐2 IgM/G, according to the manufacturer, was 10.00 AU/mL. When the IgM/IgG concentration was <10.00 AU/mL, it was regarded as non‐reactive; more than or equal to 10.00 AU/mL was regarded as positive.

### 2.5 Statistical analysis

SPSS 16.0 statistical software was used for data analysis. Diagnostic sensitivity, specificity, Youden's index (YI), positive predictive value (PPV), and negative predictive value (NPV) were used to evaluate the diagnostic value of the test. Counting data are expressed as a rate. The data of variables with non‐normal distributions are expressed by the median, and Mann‐Whitney *U* tests were used for comparisons. A *P* value < .05 was considered statistically significant.

## 3 RESULTS

### 3.1 General information about the subjects

According to the guideline of diagnosis and treatment of COVID‐19, 15 cases (8 men and 7 women) with typical epidemiological histories and clinical characteristics along with positive results of SARS‐CoV‐2 nucleic acid detection were placed in the COVID‐19 group. The 50 cases of patients (31 men and 19 women) with negative nucleic acid results were used as the control group. In the COVID‐19 group, the patients were 25‐87 years old, with an average age of 44.53 ± 16.92 years. In the control group, the patients were 4‐72 years old, with an average age of 40.44 ± 17.33 years. There was no significant difference in the age distribution or sex ratio between the two groups (*P* = .621, *P* = .393).

In the COVID‐19 group, there were 3 cases of SARS‐CoV‐2 infection with hypertension, 1 case with hyperlipidemia, 1 case with cholecystitis, and 1 case with gallstones. According to the guidelines, 1 of 15 cases was classified as mild symptoms, 12 of 15 cases were classified as common severity of symptoms, and 2 of 15 cases were in severe or critical illness conditions. Only fever (4/15), fever with cough (6/15), fever with a sore throat (1/15), fever with muscle aches (1/15), fever with palpitations (1/15), and a sore throat with a dry cough (2/15) were the first onset symptoms. In our study, there were no patients with an asymptomatic infection. The shortest hospital stay was 4 days, the longest was 31 days, and the average hospital stay was 13.60 ± 7.85 days.

### 3.2 Detecting the concentration of SARS‐CoV‐2 IgM and IgG by CLIA (quantitative)

We found in the control group the serum concentrations of SARS‐CoV‐2 IgM and SARS‐CoV‐2 IgG were 0.46 AU/mL and 0.74 AU/mL, respectively. The medians of SARS‐CoV‐2 IgM and IgG in the COVID‐19 group were 17.86 AU/mL and 69.23 AU/mL, respectively (Table 1). The concentrations of IgM and IgG in the COVID‐19 group were much higher than in the control group (*P* < .001, *P* < .001).

**Table 1:** SARS‐CoV‐2 IgM and IgG concentrations in the COVID‐19 and control groups (AU/mL)

Group	Antibody	Median	Minimum	Maximum	Cases
Control	IgG	0.74	0.10	7.55	50
	IgM	0.46	0.07	3.16	
COVID‐19	IgG	69.23	1.86	179.36	15
	IgM	17.86	0.31	218.61	

### 3.3 The diagnostic values of SARS‐CoV‐2 IgM and IgG

**Figure 1:**
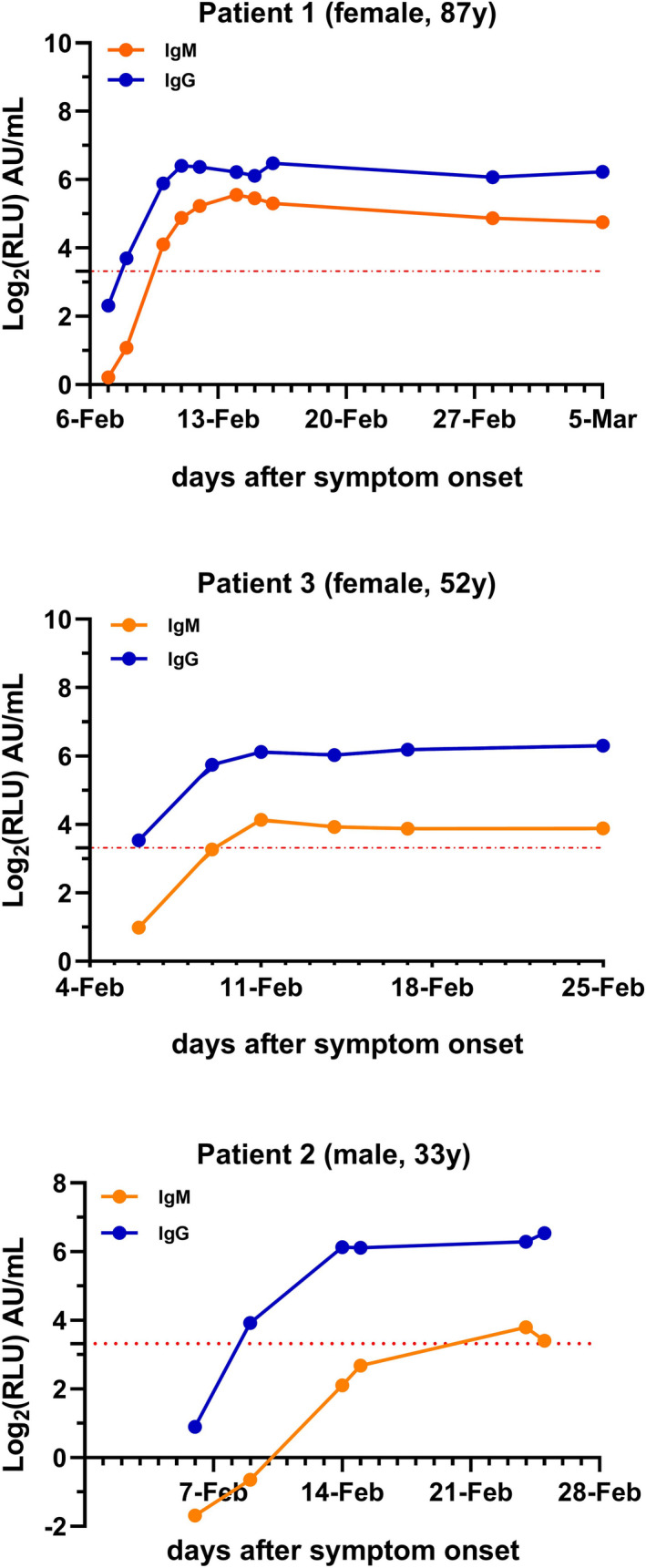
A relatively complete time course of the IgM and IgG response was observed in 3 patients. IgG is depicted in blue, and IgM is depicted in orange. The *x*‐axis shows the synchronous date of detection of IgM and IgG from the day of symptom onset. The *y*‐axis shows the log_2_ of IgM and IgG concentrations (Log_2_RLU). The red dotted line is the cutoff value y = Log_2_10

According to the cutoff value recommended by the manufacturer (cutoff = 10 AU/mL), the sensitivity, specificity, Youden index (YI), positive predictive value (PPV), and negative predictive value (NPV) of IgM were 60%, 100%, 60%, 100%, and 89.29%, respectively, and 86.67%, 100%, 86.67%, 100%, and 96.15%, respectively, for IgG. The diagnostic sensitivity of IgM was much lower than that of IgG (60% vs 86.67%). When we followed the serological courses of the COVID‐19 patients, we found 40% (6/15) COVID‐19 patients of IgM seroconversion was later than that of IgG (cutoff value = 10 AU/mL) (Figure 1).

Considering the high diagnostic efficiency of IgG, we chose the day on which the SARS‐CoV‐2 IgG was close to 10.00 AU/mL. Then, we collected IgM data on the same day to select new cutoff values. When the cutoff value of IgM was 1.83 AU/mL, we obtained the maximum YI. The sensitivity, specificity, YI, PPV, and NPV were 93.33%, 98%, 91.33%, 93.33%, and 98%, respectively. The sensitivity, YI, and NPV were much better with the new cutoff (Tables 2 and 3).

**Table 2:** SARS‐CoV‐2 IgM and IgG (IgM/IgG cutoff = 10.00 AU/mL)

IgM	Nucleic acid detection		Cases	IgG	Nucleic acid detection		Cases
	Positive	Negative			Positive	Negative	
Positive	9	0	9	Positive	13	0	13
Negative	6	50	56	Negative	2	50	52
Cases	15	50	65	Cases	15	50	65

**Table 3:** The diagnostic value of SARS‐CoV‐2 IgM and IgG

Antibody	Cutoff value	AUC	Sensitivity (%)	Specificity (%)	YI (%)	PPV (%)	NPV (%)
IgM	10.00	0.978	60	100	60	100	89.29
	1.83		93.33	98	91.33	93.33	98
IgG	10.00	1.00	86.67	100	86.67	100	96.15

### 3.4 SARS‐CoV‐2 IgM and IgG seropositive after symptoms onset

We found the shortest time for IgM to become positive was 1.5 days after the onset of symptoms, and the longest time was 7 days. The shortest time to become IgG positive was 2 days after symptom onset, and the longest time was 8 days. The positive rates of IgM and IgG in total were 96.36% and 94.55%, respectively. At 8‐14 days after symptom onset, the seropositive rates of IgM and IgG both reached 100%, which was maintained to the fourth week after symptom onset.

## 4 DISCUSSION

In this study, we dynamically detected the concentration variations of IgM and IgG in patients infected with SARS‐CoV‐2 and explored their diagnostic value for COVID‐19. Compared with nucleic acid detection, antibody detection greatly shortens the sample detection time, and it is less complicated to perform. The chemiluminescence method is a quantitative serological antibody detection assay, which has high sensitivity and specificity. Testing for antibodies can reflect whether the patient is in a state of acute infection. Convalescent plasma or hyper‐immune immunoglobulin from patients that contains significant antibody titers can likely reduce the viral load and disease mortality.^17^, ^18^ Using CLIA for quantitative detection of SARS‐CoV‐2 antibodies would be helpful in the diagnosis of COVID‐19.

In our study, we found that the median IgM in COVID‐19 patients was 17.86 AU/mL, and the median of IgG was 69.23 AU/mL after symptom onset. According to the cutoff values from the manufacturer's instructions, the sensitivity of IgM was 60%, and 6 of 15 COVID‐19 patients could not be recognized by this cutoff. Another researcher also used the Yhlo CLIA diagnostic test kit and found the sensitivity of IgM and IgG to diagnose COVID‐19 was 70.24% and 96.10%, respectively.^19^ Those results showed the same problems as our data.

In order to improve the diagnostic sensitivity of IgM, we selected different cutoff values for IgM. Considering the high diagnostic efficiency of IgG, we chose the day on which the SARS‐CoV‐2 IgG was close to 10.00 AU/mL and then collected the IgM data on the same day for setting the new cutoff values. When the IgM cutoff value was 1.83 AU/mL, we achieved the maximum test efficiency of IgM, and its sensitivity was 93.33%, which is high enough to make it beneficial for diagnosing COVID‐19.

Dynamically detecting the concentrations of IgM and IgG after symptom onset, we found the shortest times for patients to become IgM and IgG seropositive were 1.5 and 2 days, respectively, and the longest times were 7 and 8 days, respectively. The positive rates of IgM and IgG both increased as the time increased, and 8‐14 days after onset of the disease, the seropositive rates of IgM and IgG both reached 100%, which was then maintained to the fourth week after symptom onset.

This finding indicates that SARS‐CoV‐2 infection can be ruled out if antibodies against SARS‐CoV‐2 are still undetectable by 14 days of symptom onset. Our findings may suggest that for patients who missed the ideal nucleic acid sampling window but have typical COVID‐19 symptoms or chest imaging abnormalities, that testing for IgM and IgG antibodies would be sufficient to confirm the COVID‐19 diagnosis. It is worth noting that approximately 13.33% (2/15) of the patients who had symptoms and were confirmed to be positive by RT‐PCR were found to be negative by both the IgM and IgG antibody tests. One case was tested 1 day after symptom onset, and the other case was tested 6 days after symptom onset. These negative results are probably due to individual patient differences in the amounts of antibodies being produced. Although we cannot absolutely rely on CLIA testing for a COVID‐19 diagnosis, we believe that CLIA combined with RT‐PCR can provide more accurate COVID‐19 diagnoses.

There are several limitations to our study. First of all, this small sample size must be discreetly analyzed. Secondly, this antibody detection kit was not available at the beginning of the outbreak of SARS‐CoV‐2 in China, and some patients who were discharged without antibody testing could not be enrolled in our study. Thirdly, as of the last date of this study (March 5), the IgM level never returned to seronegative in the COVID‐19 patients. At the same time, we do not know how long the IgG seropositivity will last. Therefore, we have not completely analyzed the dynamic changes of SARS‐CoV‐2‐specific antibodies. In the future, we will continue to follow up on the changes of SARS‐CoV‐2‐specific IgG/IgM in discharged COVID‐19 patients.

In conclusion, the IgM cutoff value 1.83 AU/mL for the diagnosis of COVID‐19 was much better than the cutoff provided by the manufacturer. SARS‐CoV‐2 infection can be ruled out if antibodies against SARS‐CoV‐2 are still undetectable by 14 days of symptom onset.
